# Astaxanthin Inhibits Expression of Retinal Oxidative Stress and Inflammatory Mediators in Streptozotocin-Induced Diabetic Rats

**DOI:** 10.1371/journal.pone.0146438

**Published:** 2016-01-14

**Authors:** Po-Ting Yeh, Hsin-Wei Huang, Chung-May Yang, Wei-Shiung Yang, Chang-Hao Yang

**Affiliations:** 1 Department of Ophthalmology, National Taiwan University Hospital, Taipei, Taiwan; 2 Graduate Institute of Pathology, College of Medicine, National Taiwan University, Taipei, Taiwan; 3 Department of Ophthalmology, Taipei Medical University-Wan Fang Hospital, Taipei, Taiwan; 4 Department of Ophthalmology, College of Medicine, National Taiwan University, Taipei, Taiwan; 5 Graduate Institute of Clinical Medicine, College of Medicine, National Taiwan University, Taipei, Taiwan; 6 Department of Internal Medicine, National Taiwan University Hospital, Taipei, Taiwan; Medical University of South Carolina, UNITED STATES

## Abstract

**Purpose:**

We evaluated whether orally administered astaxanthin (AST) protects against oxidative damage in the ocular tissues of streptozotocin (STZ)-induced diabetic rats.

**Methods and Results:**

Fifty 6-week-old female Wistar rats were randomly assigned to receive an injection of STZ to induce diabetes (n = 40) or to remain uninduced (n = 10). The diabetic rats were randomly selected into four groups and they were separately administered normal saline, 0.6 mg/kg AST, 3 mg/kg AST, or 0.5 mg/kg lutein daily for eight weeks. Retinal functions of each group were evaluated by electroretinography. The expression of oxidative stress and inflammatory mediators in the ocular tissues was then assessed by immunohistochemistry, western blot analysis, ELISA, RT-PCR, and electrophoretic mobility shift assay (EMSA). Retinal functions were preserved by AST and lutein in different levels. Ocular tissues from AST- and lutein-treated rats had significantly reduced levels of oxidative stress mediators (8-hydroxy-2'-deoxyguanosine, nitrotyrosine, and acrolein) and inflammatory mediators (intercellular adhesion molecule-1, monocyte chemoattractant protein-1, and fractalkine), increased levels of antioxidant enzymes (heme oxygenase-1 and peroxiredoxin), and reduced activity of the transcription factor nuclear factor-kappaB (NF-κB).

**Conclusion:**

The xanthophyll carotenoids AST and lutein have neuroprotective effects and reduce ocular oxidative stress, and inflammation in the STZ diabetic rat model, which may be mediated by downregulation of NF-κB activity.

## Introduction

Diabetes are metabolic disorders characterized by dysregulation of blood glucose levels. Diabetic retinopathy is the most serious sight-threatening complication of diabetes [1, 2]. Although our understanding of the pathogenesis of diabetic retinopathy has improved, and medical and surgical treatments have advanced, the long-term outcome of diabetic retinopathy remains poor. Therefore, there is a continuing need to search for a new modality for preventing and treating this debilitating complication.

The hyperglycemia that occurs in diabetes increases the production of reactive oxygen species (ROS) and depletes cellular antioxidant defense capacities, resulting in enhanced oxidative stress. Chronic oxidative stress is considered one of the primary causes of diabetic retinopathy [3–7]. The retina has a high content of unsaturated fatty acids and high oxygen uptake, which increases lipid oxidation and ROS production. This is commonly thought to make the retina more vulnerable than any other tissue to oxidative stress damage [8, 9].

Inflammation may also play a key role in the development and progression of diabetic retinopathy [10, 11]. ROS are strong stimulators of the transcription factor nuclear factor kappa B (NF-κB), which increases the transcription of inflammatory cytokines and chemokines as well as enzymes responsible for nitric oxide and prostaglandin E2 synthesis. All of these factors are involved in the pathogenesis of diabetic retinopathy [12–15]. Antioxidants have long been known to inhibit inflammatory responses. In animal models of diabetic retinopathy, antioxidants inhibit NF-κB activity, and reduce leukostasis and leukocyte expression of inducible nitric oxide synthase [16]. Moreover, antioxidants can inhibit the formation of cell-free capillaries and generation of pericyte ghosts in diabetic rats [17, 18]. In addition, antioxidants inhibit the formation of ROS and increase the capabilities of the antioxidant defense enzyme system [19, 20]. Therefore, antioxidants might diminish the biologic damage of oxidative stress in the retina, abate the level of inflammation, and arrest the progression of diabetic retinopathy.

Astaxanthin (AST) and lutein both are xanthophyll family of hydroxycarotenoids which contain several double bonds. They could scavenge ROS to be powerful biological antioxidants and anti-inflammatory agents [21–24]. AST is present in many organisms and is especially rich in seafood such as salmon, trout, sea bream, shrimp, lobster, fish eggs, and algae. Lutein is a yellow crystal that is found in some vegetables, such as kale, spinach, and broccoli. AST removes single oxygen atoms, eliminates free radicals, and prevents and terminates peroxidation chain reactions by transferring the chemical energy into heat removal [25, 26]. AST is a more potent antioxidant than other carotenoids, including lutein, β-carotene, canthaxanthin, and zeaxanthin. Also several reports demonstrated AST could be safely taken by human and rats [27, 28]. Although AST is not naturally present in the human retina, it easily crosses the blood-brain barrier and subsequently protects retinal ganglion cells from oxidative damage [29]. Lutein has been proven to reduce oxidative stress in the retina and inhibit the downstream pathological signals in the diabetic retinopathy animal model [24]. In addition, the antioxidant activities of AST is more potent than lutein [30], however, AST has never been reported that it is benefit for reducing oxidative stress in eyes of diabetes. For these reasons, we hypothesized that AST is a powerful antioxidant that is superior to lutein in diabetes. AST may protect the retina from the various oxidative stresses and inflammatory insults that accompany diabetes.

In this study, we evaluated the potential protective effects of AST against diabetes-induced retinal damage in streptozotocin (STZ)-induced diabetic rats. We examined the effects of AST on the production of oxidative stress mediators, the activation of NF-κB, and the expression of downstream inflammatory mediators in the ocular tissues of diabetic rats.

## Materials and Methods

### Ethics statement

This study was carried out in strict accordance with the recommendations in the Guide of the Association for Research in Vision and Ophthalmology Statement for the Use of Animals in Ophthalmic and Vision Research. The protocol was approved by the Institutional Animal Care and Use Committee of National Taiwan University College of Medicine and College of Public Health (Permit Number: 20110211). All surgery was performed under sodium pentobarbital anesthesia, and all efforts were made to minimize suffering.

### Animals groups and induction of diabetes

Fifty female Wistar rats, aged 6 to 8 weeks and weighing 200 to 250 g, were obtained from the Animal Resource Center, College of Medicine, National Taiwan University. The rats were randomly assigned to five groups (n = 10 per group): (1) nondiabetic controls (referred to as Control group in the results section), and four groups of STZ-induced diabetic rats that received (2) no supplementation (Diabetes group), (3) a high dose of AST (3 mg/kg/day; AST High group), (4) a low dose of AST (0.6 mg/kg/day; AST Low group), or (5) lutein (0.5 mg/kg/day; Lutein group).

Diabetes was induced by intraperitoneal injection of 55 mg/kg STZ (Sigma, St. Louis, MO) dissolved in citrate buffer, pH 4.5. The Control group was injected with the same volume of citrate buffer. Seventy-two hours after STZ injection, blood glucose levels were measured and found to be >250 mg/dL, indicating the successful induction of diabetes.

The rats were administered the appropriate doses of AST and lutein (AST High, AST Low, and Lutein groups) or an equivalent volume of normal saline (Diabetic group), every day for the next 8 weeks via an intragastric feeding tube. The Control group received no intervention. Body weights and blood glucose levels were recorded 72 h after injection of STZ or vehicle and again at the end of 8 weeks.

### Electroretinogram (ERG)

The ERG was performed on all rats at day 0 and day 56. The procedures of ERG had been described previously [31, 32]. In brief, rats were kept in the dark room for 12 h before performing the ERG. All manipulations were done with dim red light illumination. After being anesthetized, the rats were placed on a heating pad and a recording electrode was placed on the cornea after application of 0.5% methyl cellulose. A reference electrode was attached to the shaven skin of the head and a ground electrode clipped the rat’s tail. A single flash light (duration, 100 ms) 30 cm from the eye was used as the light stimulus. Responses were amplified with a gain setting ±500 μV and filtered with low 0.3 Hz and high 500 Hz from an amplifier. The pattern of the a- and b-wave was recorded. The fold of b-wave ratio was defined as b-wave amplitude of right eye at day 56/b-wave amplitude of right eye at day 0.

### Tissue preparation

After 8 weeks, the rats were euthanized by intraperitoneal injection of a lethal dose of pentobarbital. The abdominal cavity was opened and blood was collected by direct puncture of the descending aorta. Sera were prepared and frozen until use. The eyes were harvested and the retina was isolated under a microscope and immediately frozen in liquid nitrogen. The aqueous and vitreous humors were also collected for further analysis.

### Histopathologic examination

The procedures had been described in previous study [33]. In brief, histopathological specimens were obtained at 0.5 mm dorsal and ventral from the optic disc and evaluated by a light microscope (Olympus BX 52, Tokyo, Japan). Color micrographs were captured at ×400 magnification with a digital color camera for microscope (Olympus DP 72, Tokyo, Japan). The number of cells in the ganglion cell layer (GCL) was calculated using the linear cell density (cells per 100 μm). The thicknesses of total retina (between the inner limiting membrane and pigment epithelium), inner plexiform layer (IPL), inner nuclear layer (INL), and the combined thickness of outer plexiform and outer nuclear layers (pooled as the outer retinal layers, ORL) were measured. Each specimen was measured at three random sites and three specimens were done. The results of the five groups were statistically analyzed.

### Immunofluorescence (IF) detection of oxidative stress and inflammatory mediators

IF was carried out by simultaneously blocking and permeabilizing sections with 0.2% Triton in PBS containing 5% goat serum for 1 hour at room temperature, incubating with primary antibodies diluted in blocking solution overnight at 4°C, and incubating with the appropriate fluorescent secondary antibodies (all diluted 1:1000) in blocking solution for 3 hours at room temperature. Nuclei were counterstained with DAPI. Primary antibodies included mouse anti-rat 8-hydroxy-2'-deoxyguanosine (8-OHdG) (JaICA Inc., catalog no. MOG-020P), mouse anti-rat nitrotyrosine (Abcam Inc., catalog no. Ab-7048), and mouse anti-rat acrolein (Abcam Inc., catalog no. Ab-48501).

The following formula was used for the densitometric quantitation of acrolein, nitrotyrosine, and 8-OHdG immunofluorescence, as previously described [34].

Immunostaining index=Σ[(X−threshold)×area(pixels)]/total cell number

Where X is the staining density indicated by a number between 0 and 256 in grayscale, and X is more than the threshold. Briefly, digitized color images were obtained as PICT files. PICT files were opened in grayscale mode using NIH image, ver. 1.61. Cell numbers were determined using the Analyze Particle command after setting a proper threshold.

### Immunohistochemical (IHC) detection inflammatory mediators

Sections (6 μm) of 10% formalin-fixed, paraffin-embedded eye tissues were placed on slides, deparaffinized in xylene, rehydrated in a graded series of ethanol, and finally washed in PBS. Endogenous peroxidase activity was blocked by incubation of slides with 0.3% hydrogen peroxide in methanol. Sections were stained with hematoxylin & eosin (H&E) for examination of pathological changes in morphology. For analysis of inflammatory mediators, the sections were incubated overnight at 4°C with the following primary antibodies: mouse anti-rat intercellular adhesion molecule-1 (ICAM-1) (Lifespan Bioscience Inc., catalog no. LS-B1850), rabbit anti-rat monocyte chemoattractant protein-1 (MCP-1) (Abcam Inc., catalog no. Ab-7202), goat anti-rat fractalkine (FKN) (R&D Systems Inc., catalog no. AF537), and mouse anti-rat NF-κB (Santa Cruz Biotechnology, Inc., catalog no. SC-8008).

After incubation with primary antibodies, the sections were washed and incubated with the appropriate biotinylated secondary antibody to goat, mouse, or rabbit IgG, followed by incubation with an avidin-biotinylated horseradish peroxidase complex (Santa Cruz Biotechnology) and 3,3'-diaminobenzidine as the chromogenic peroxidase substrate. Immunostained sections were counterstained with hematoxylin, dehydrated, and mounted. Negative control sections were incubated with rabbit IgG (BioVision) as the primary antibody and then processed as described above. The relative staining intensities were quantified by analysis of sections with the computer imaging software Image-Pro Plus 6.0 (Media Cybernetics, Inc., Bethesda, MD)

### Protein extraction and western blot analysis

Proteins were extracted from isolated rat retinas by incubation in a lysis buffer consisting of 0.5 M Tris-HCl (pH 7.4), 1.5 M NaCl, 2.5% deoxycholic acid, 10% NP-40, 10 mM EDTA, and protease inhibitors (Complete Mini; Roche Diagnostics Corp., Indianapolis, IN). For western blot analysis, the protein samples were mixed 1:1 with Laemmli sample buffer and boiled for 5 min. Samples (equivalent to 100 μg total protein) were resolved by 10% SDS-PAGE. Separated proteins were electrophoretically transferred to polyvinylidene fluoride (PVDF) membranes (Immobilon-P; Millipore Corp., Billerica, MA) in a buffer containing 200 mM NaCl, 200 mM Tris-base, and 10 mM MgCl_2_ (pH 9.5). Nonspecific binding was blocked by incubation of membranes with 5% milk in PBS containing 0.1% Tween-20 (PBST) for 1 hour at room temperature. The blots were then incubated with the following primary antibodies diluted in 5% milk in PBST overnight at 4°C: goat anti-rat ICAM-1(1:1000 dilution; R&D Systems Inc., catalog no. AF583), goat anti-rat MCP (1:1000; Santa Cruz Biotechnology Inc., catalog no. SC-1785), goat anti-rat FKN (1:1000; R&D Systems Inc., catalog no. AF537), or mouse anti-rat β-actin (1:5000; Abcam Inc., catalog no. ab8224). After washing with PBST, the membranes were incubated with horseradish peroxidase-conjugated secondary antibody for one hour and visualized by chemiluminescence. (Pierce Biotechnology, Rockford, IL). The relative expression of proteins was quantified by densitometry scanning of blots with ImageJ 1.45e software (National Institutes of Health, Bethesda, MD).

### Quantification of ICAM-1, MCP-1, and FKN in the aqueous humors

The levels of ICAM-1, MCP-1, and FKN in the aqueous were quantified using sandwich ELISA kits (R&D Systems., catalog no. RIC100; PeproTech, Inc., catalog no. 900-K59; R&D Systems., catalog no. DY537, for ICAM-1, MCP-1, and FKN, respectively). The aqueous humor were pooled by each group. Total protein content in each sample was determined by the Bradford assay (Bio-Rad). The pooled samples were measured by 3 repeat ELISA experiments.

### Preparation of RNA and cDNA for PCR

Total RNA was extracted from the retinas with TRIzol reagent (Invitrogen-Life Technologies Inc., Gaithersburg, MD) according to the manufacturer’s protocol. One microgram of total RNA from each sample was reverse transcribed by annealing for 5 min at 65°C with 300 ng oligo(dT) (Promega, Madison, WI) and incubation with 80 U Moloney murine leukemia virus reverse transcriptase (MMLV-RT; Invitrogen-Gibco, Grand Island, NY) for 1 h at 37°C. The reaction was stopped by heating for 5 min at 90°C.

PCR was performed with gene-specific primers for heme oxygenase-1 (HO-1), peroxiredoxin (PRDX), thioredoxin (Trx), ICAM-1, MCP-1, FKN, and β-actin (Table 1). All primers were prepared by Mission Biotech (Taipei, Taiwan). The amplifications were performed with a thermocycler (MJ Research, Waltham, MA). Reactions (50 μL final volume) contained 5 μL of cDNA, 1 μL each of sense and antisense primers, 200 μM of each deoxynucleotide, 5 μL of 10× Taq polymerase buffer, and 1.25 U Taq polymerase (Promega). The cycling conditions were 95°C for 5 min followed by 28–32 cycles (see Table 1) of 94°C for 1 min, 55°C for 2 min, and 72°C for 3 min, followed by an extension for 10 min at 72°C and final cooling to 4°C. PCR products were separated by electrophoresis in 2% agarose gels containing ethidium bromide (Sigma-Aldrich) and visualized under ultraviolet light/ The product intensities were quantified by computer image analysis (Digital 1D Science; Eastman Kodak, Rochester, NY). mRNA expression levels were normalized to that of the housekeeping gene, β-actin. All experiments were repeated three times.

**Table 1:** PCR primer sequences and amplified product sizes.

Gene	Primer sequence	Product Size (bp)	Annealing Temp°C	Cycles
HO-1	5′-CACGCCTACACCCGCTACCT 5′-TCTGTCACCCTGTGCTTGAC		52	32
PRDX	5′-TGCCAGATGGTCAGTTTAAA 5′-CAGCTGGGCACACTTCCCCA		53	32
Trx	5′-CTGCTTTTCAGGAAGCCTTG 5′-TGTTGGCATGCATTTGACTT		52	32
ICAM-1	5′-CCTGTTTCCTGCCTCTGAAG 5′-CCTGGGGGAAGTACTGTTCA	830 bp	52	30
MCP-1	5′-CTACAGAAGTGCTTGAGGTGGTTG 5′-CTGGGCCTGTTGTTCACAGTTGC	436 bp	52	30
FKN	5′-CCTCGGCATGACGAAATGCA 5′-AGGCCCTGGAGATTTCTCTG	703 bp	52	28
β-actin	5′-CTGGAGAAGAGCTAGAGCTG 5′-AATCTCCTTCTGCATCCTGTC	246 bp	52	22

### Nuclear protein extraction and electrophoretic mobility shift assay (EMSA)

Retina nuclear proteins were extracted with NE-PER (Nuclear and Cytoplasmic Extraction Reagent; Pierce, Rockford, IL, USA) according to the manufacturer’s instructions. Protein was quantified by the BCA method (Bio-Rad). All extracts were stored at -80°C until use.

The methods of EMSA had been reported in previous studies [35, 36]. In brief, EMSAs were performed using a Light Shift Chemiluminescent EMSA Kit (Pierce, Rockford, IL, USA). To measure the capacity of the nuclear extract proteins to interact with consensus NF-κB–binding sequences, 10 μg of each nuclear extract was incubated with a biotinylated DNA duplex (5′-AGTTGAGGGGACTTTCCCAGGC-3′ and 3′-TCAACTCCCCTGAAAGGGTCCG-5′) containing a putative NF-κB consensus binding site [37]. The samples were incubated for 30 min in a total volume of 20 μL buffer consisting of 10 mM Tris-Cl, pH 7.5, 1 mM MgCl_2_, 50 mM NaCl, 0.5 mM DTT, 0.5 mM EDTA, 4% glycerol, and 2 μg of polydeoxyinosinic-deoxycytidylic acid. The specificity of DNA–protein binding was confirmed by the addition of a 100-fold molar excess of unlabeled oligonucleotide to the extract 10 min before the addition of the biotinylated probe. After the reaction, the samples were subjected to 6% PAGE and transferred to a nylon membrane, which was cross-linked for 15 min on a UV transilluminator at 312 nm. Biotin-labeled DNA–protein complexes were detected by chemiluminescence, and the membranes were exposed to X-ray films.

### Statistical analysis

Data are expressed as the mean ± SD. Differences between all groups were analyzed by one-way analysis of variance followed by Bonferroni’s test for multiple comparisons, if appropriate. All of the statistical analyses were performed using STATA 8.2 software (StataCorp LP, College Station, TX). A *P* value of less than 0.05 was considered statistically significant. A value of *p* < 0.05 was considered statistically significant.

## Results

### Body weights and blood sugar levels of experiment rats

The body weights and blood sugar levels of rats at the beginning and at the end of the experiments are presented in Table 2. After diabetes induction and feeding for 8 weeks, animals in the normal Control, AST High, and AST Low groups gained more weight than the Diabetes and Lutein groups. However, the differences in body weights among the five groups did not reach statistical significance (*p* = 0.88). Animals in the four STZ-injected groups had significantly higher glucose levels than the Control group, both immediately after STZ injection and at the end of 8 weeks (*p* < 0.05; Table 2). There were no significant differences in the blood sugar levels among the four experimental groups.

**Table 2:** Body weights and blood sugar levels in the study groups.

Groups	Control	Diabetes	Diabetes + High AST	Diabetes + Low AST	Diabetes + Lutein
**(A)**					
Body Weight (g)	247.8 ± 18.9	229.4 ± 23.0	226.6 ± 10.5	229.7 ± 12.6	226.6 ± 21.2
Blood Sugar (mg/dL)	115.3 ± 27.1	486 ± 54.8*	479 ± 62.6*	482 ± 56.8*	491 ± 33.9*
**(B)**					
Body Weight (g)	276.7 ± 21.8	247.7 ± 45.7	255.5 ± 14.4	259.5 ± 18.5	247.0 ± 17.5
Blood Sugar (mg/dL)	131.7 ± 27.6	598.3 ± 27.1*	568.0 ± 40.6*	597.8 ± 48.2*	550.6 ± 66.8*
**(B)-(A)** Body weight (g)	28.9 ± 33.6	20.1 ± 26.1	28.9 ± 16.8	29.8 ± 16.6	20.4 ± 17.4

(A), 72 h after STZ injection; (B), 8 weeks after STZ injection.

Values are expressed as the mean ± SD of 10 rats per group.

* *p* < 0.01 versus Control group

### The effects of AST and lutein on ERG

**Fig 1:**
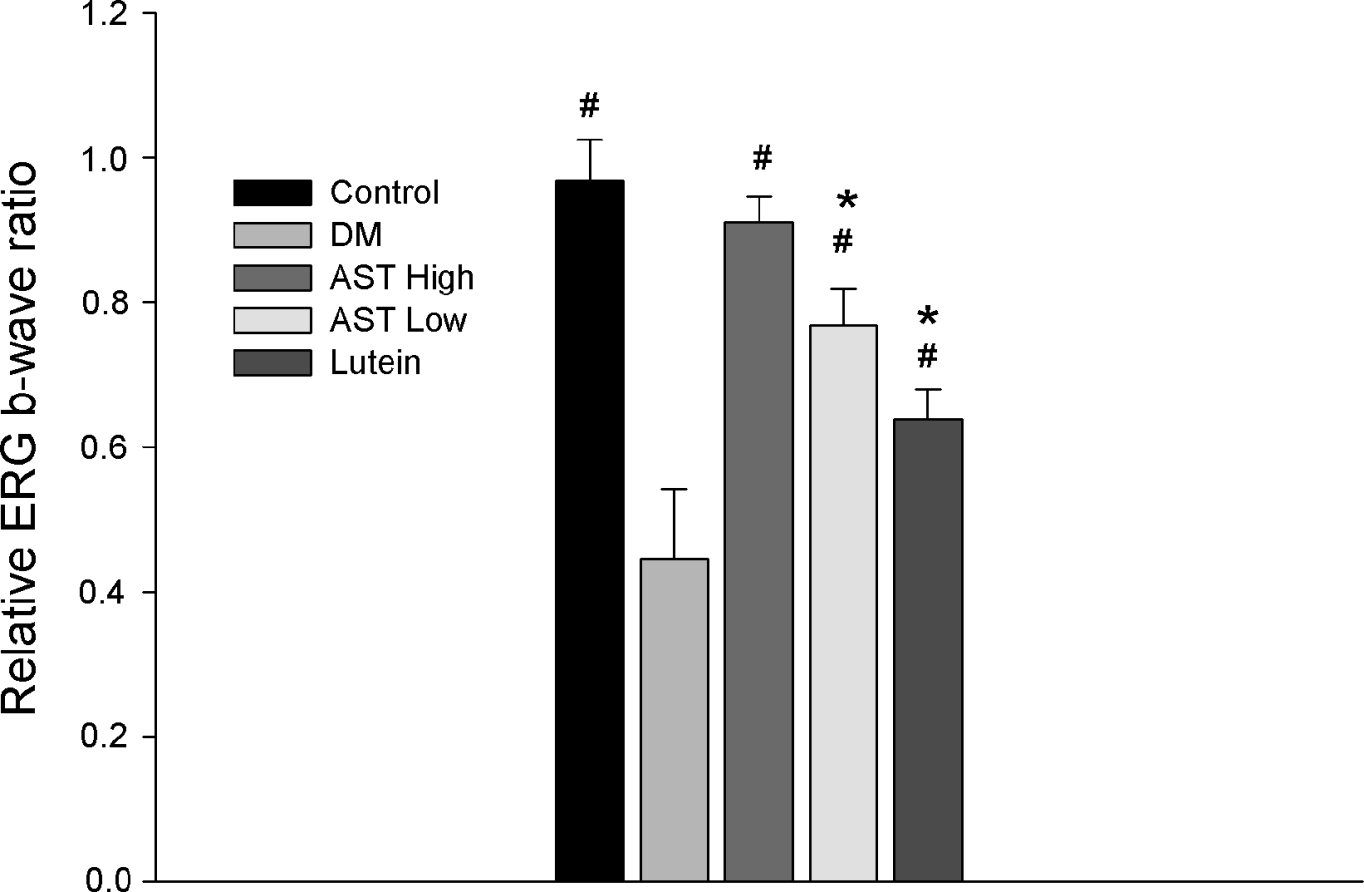
Evaluation of functional changes of the retina by electroretinography (ERG). The ERG were performed on untreated rats (Control) or STZ-induced diabetic rats treated for 8 weeks with normal saline (Diabetes), 3 mg/kg AST (AST High), 0.6 mg/kg AST (AST Low), or 0.5 mg/kg lutein (Lutein). The relative b-wave ratio was significantly decreased in the Diabetes, AST Low and Lutein groups compared with the Control group. The relative b-wave ratio in AST High group had no significant difference from Control group. The ratio in AST High group was significantly higher than that in the AST Low and the Lutein groups (*p* = 0.046 and *p* < 0.001). The data are expressed as the mean ± SD in 4 rats for each group (bar graph). *, *p* < 0.05 compared with the control group. #, *p* < 0.05 compared with the Diabetes group. Differences among groups were analyzed by one-way analysis of variance followed by Bonferroni’s test for multiple comparisons.

The relative b-wave ratio was approximately equal to 1 in the normal controls. For the diabetic groups, the b-wave ratios were significantly higher in groups treated with supplements than the group without supplements. In addition, only the high-dose AST group had no significant difference between control group in b-wave ratio (*p* = 1.0). Otherwise, the diabetic rats without supplements group, low-dose AST group or lutein group, the ratios were all significantly decreased (*p*<0.05 in all paired comparisons with control rats). Notably, in rats treated with high-dose AST, the relative b-wave ratio was higher than that in the lutein and the low-dose lutein groups (*p* = 0.046 and *p* < 0.001) (Fig 1).

### Histology examination, IF and IHC detection of oxidative stress mediators in retinas

**Fig 2:**
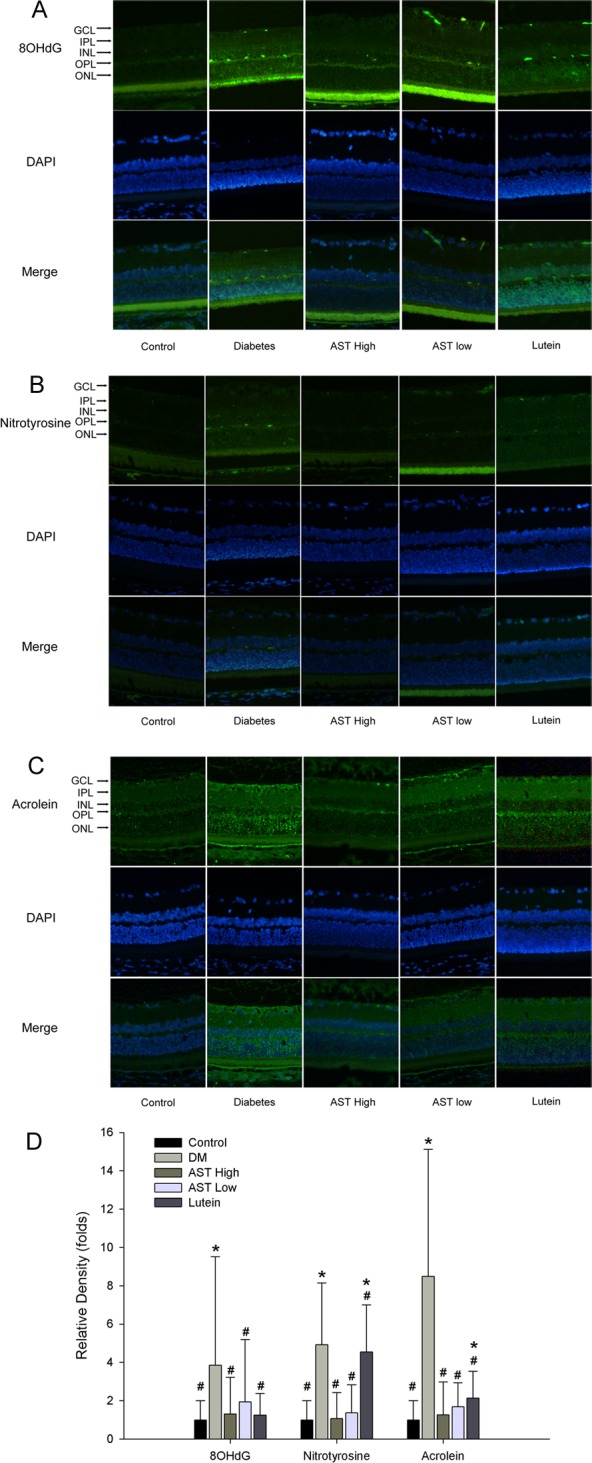
Immunofluorescence staining of oxidative stress mediators in retinas. Retinal sections were prepared from Control, Diabetes, AST High, AST Low, or Lutein groups. Retinal oxidative damages were evaluated by immunofluorescence staining of (A) 8-hydroxy-2'-deoxyguanosine (8-OHdG) (B) nitrotysine, and (C) acrolein (original magnification 200×). (D) The relative density of immunostaining was defined as immunostaining index of control group. For quantitation of immunostaining, we first determined the immunostaining index, which could be measured and calculated from the following formula: Σ [(immunostaining density-threshold) × area (pixels)] / total cell number. Treatment with AST and lutein decreased the staining for nitrotysine, acrolein and 8-OHdG in the retinas compared with Diabetes group but the staining density of nitrotyrosine and acrolein in lutein group were significantly higher than control group. Data are presented as the mean ± SD. *, p < 0.05 versus the Control group; #, p < 0.05 versus the Diabetes group.

The total retinal thickness, number of ganglion cells, the thickness of IPL, INL and ORL were significant decreased in diabetic group compared to control and the other study groups (*p*<0.05) (Table 3). We examined the expressions of 8-OHdG, nitrotyrosine, and acrolein in retina as indicators of oxidative stress induced by peroxidation of DNA, protein, and lipid, respectively (Fig 2A, 2B and 2C). The most intense staining of 8-OHdG, nitrotyrosine, and acrolein was observed in the diabetes group, and the intensities were all reduced significantly in AST High, AST Low, and Lutein groups compared with diabetes group.

**Table 3:** Thickness of the retinal layers and ganglion cell layer (GCL) cell counts.

	Thickness (μm)				GCL cell counts (per 100 μm)
	Total retina	IPL	INL	ORL	
Control	171.3±1.4^#^	52.6±1.3^#^	26.0±1.0^#^	42.8±0.7^#^	11. 9±1.2^#^
Diabetes	148.7±2.3^*^	40.3±1.6^*^	20.5±1.2^*^	35.3±1.6^*^	7.4±0. 9^*^
AST high	170. 5±1.8^#^	52.1±1.7^#^	24.8±0.7^#^	42.3±0.8^#^	11. 7±1.0^#^
AST low	157.6±1.2^*^^#^	45.7±1.2^*^^#^	22.6±1.3^*^^#^	40.5±1.3^*^^#^	8.8±0.8^*^^#^
Lutein	156.7±1.6^*^^#^	45.25±1.5^*^^#^	21.4±0.9^*^	39.8±1.5^*^^#^	8. 9±0.8^*^^#^

INL, inner nuclear layer; IPL, inner plexiform layer; ORL, outer retinal layers indicated the combined thickness of outer plexiform and outer nuclear layers.

*, *p* < 0.05 versus the Control group

#, *p* < 0.05 versus the Diabetes group

IF staining of each reporter molecule was quantified with Image-Pro plus software and the results are presented graphically in Fig 2D. The expression of 8-OHdG, nitrotyrosine, and acrolein was highest in the Diabetes group and the expression of all three molecules was significantly reduced in the AST High and AST low groups (*p* < 0.05). In contrast, staining of nitrotyrosine and acrolein, but not nitrotyrosin, was significantly decreased in the Lutein group (*p* < 0.005).

### Expression of inflammatory mediator mRNA levels in retinas

**Fig 3:**
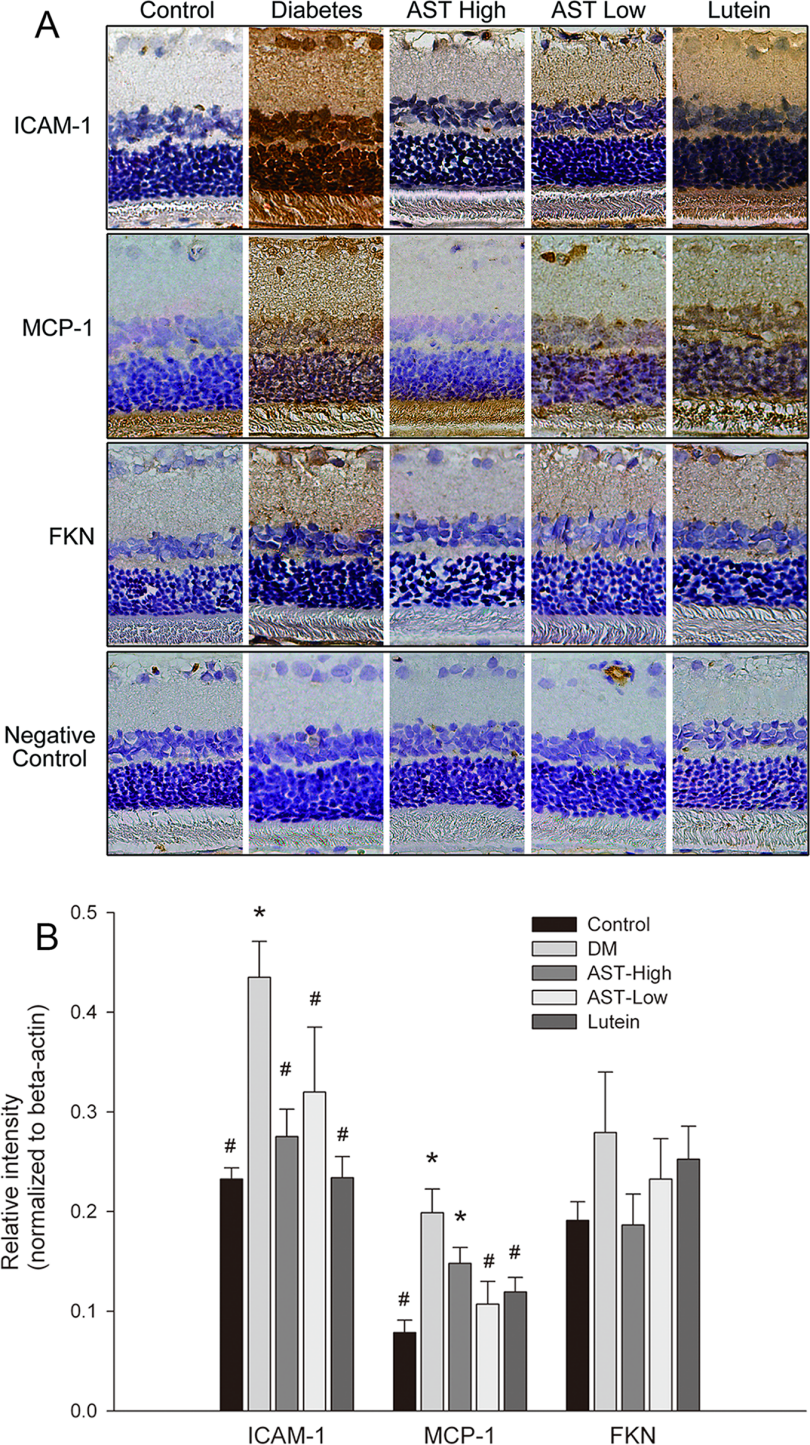
mRNA levels of inflammatory mediators in retinas. (A) RNA was subjected to RT-PCR using gene-specific primers for ICAM-1, MCP-1, FKN, and β-actin. (B) mRNA expression of ICAM-1, MCP-1, and FKN, normalized to the expression of β-actin. Data are presented as the mean ± SD. *, *p* < 0.05 versus the Control group; #, *p* < 0.05 versus the Diabetes group.

We measured the levels of ICAM-1, MCP-1, and FKN mRNA expression in each group by semi-quantitative RT-PCR (Fig 3A and 3B). In our results, ICAM-1, MCP-1, and FKN mRNA levels were significantly elevated in the retinas of the Diabetes group compared with the healthy Control group. In study groups, the mRNA levels of ICAM-1 and MCP-1were decreased in the AST High, AST Low and lutein-fed animals compared with the animals in Diabetic group. The FKN expression was significantly reduced only in AST High group compared with Diabetic group.

### Effect of AST and lutein on expression of inflammatory proteins in retinas

**Fig 4:**
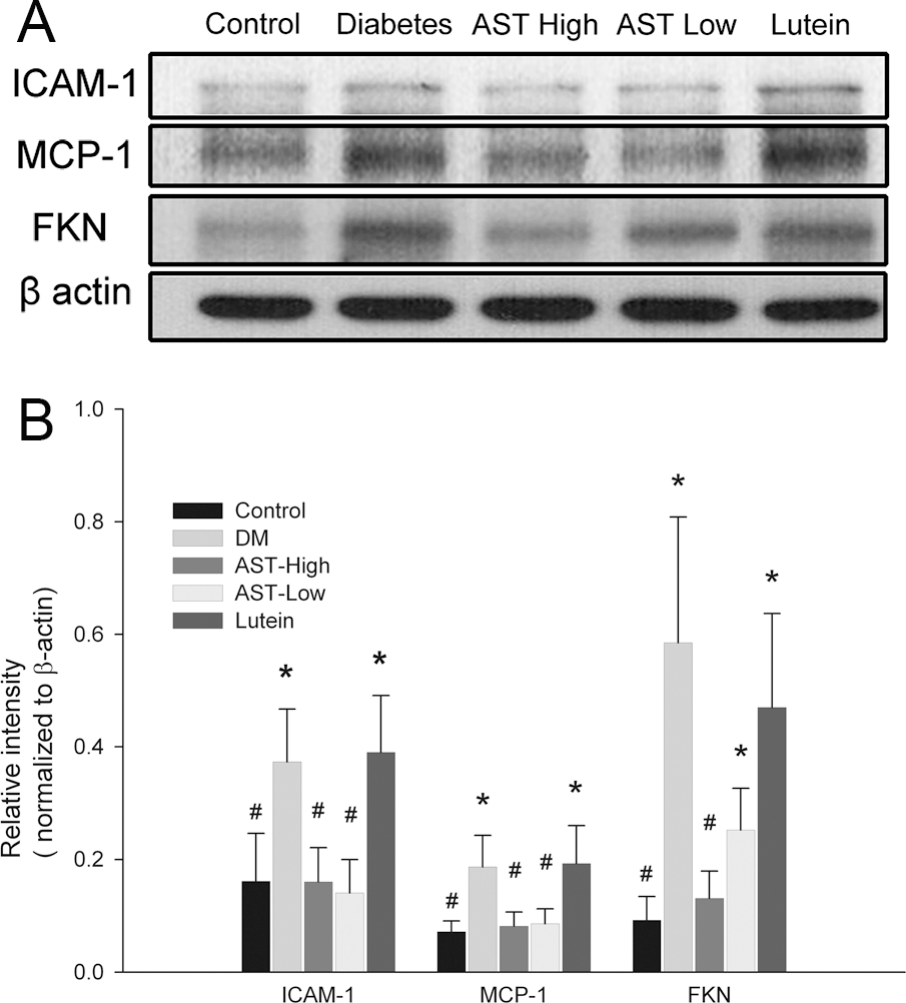
Western blot analysis of ICAM-1, MCP-1, and FKN protein expression in retinas. Retinal cell extracts were prepared from animals treated as described in Fig 1. (A) Blots were probed with antibodies specific for ICAM-1, MCP-1, and FKN. β-actin was probed as a loading control. (B) The relative intensities of the bands in (A) were determined by ImageJ software and normalized to the expression of β-actin. Data are presented as the mean ± SD. *, *p* < 0.05 versus the Control group; #, *p* < 0.05 versus the Diabetes group.

To examine the effects of AST and lutein on ICAM-1, MCP-1 and FKN expression, we analyzed the protein levels by western blotting of whole retinal extracts. The estern blotting revealed significant elevation of ICAM-1, MCP-1, and FKN protein expression in the retinas of the Diabetic group compared with the Control group (Fig 4A). Quantitation of the relative band intensities showed that the retinas from the AST High group contained significantly lower levels of all three inflammatory proteins than the Diabetes group (*p* < 0.05), and MCP-1 and ICAM-1 expression were also reduced in the AST Low group (*p* < 0.05). Although FKN expressions were reduced in the retinas of the AST Low and Lutein group compared with the Diabetes group, the difference did not reach statistically significant level (Fig 4B).

### IHC detection of inflammatory mediators in retinas

**Fig 5:**
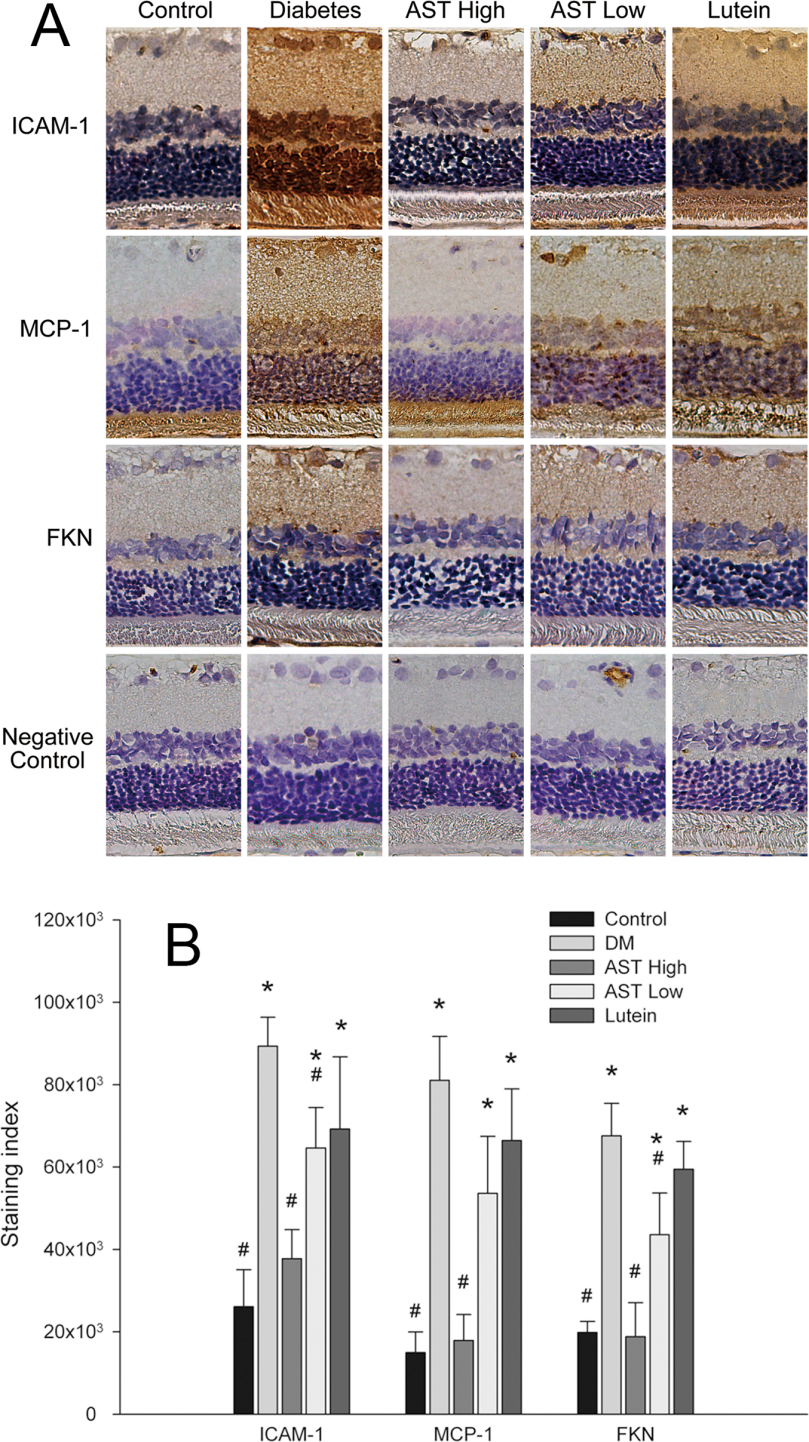
IHC staining of inflammatory mediators in retinas. Retinal sections were prepared from animals by each group. (A) Photomicrographs show immunocytochemical localization of ICAM-1, MCP-1, and FKN (original magnification 400×). (B) Images in (A) were quantified with Image-Pro software. Data are presented as the mean ± SD. *, *p* < 0.01 versus the Control group; #, *p* < 0.01 versus the Diabetes group.

We examined the expression of three NF-κB–induced inflammatory molecules, ICAM-1, MCP-1, and FKN, in the retinas and the photomicrographs of IHC staining were shown in Fig 5A.

After quantification of the positively staining cells in the images (Fig 5B), ICAM-1, MCP-1, and FKN were expressed at significantly higher levels in the Diabetic group than in Control group and AST High group. ICAM-1 and FKN levels were significantly lower in AST Low group than in the Diabetes group (*p* < 0.05). All three inflammatory protein staining were reduced in Lutein group compared with Diabetic group, but they did not meet the significant level. In contrast, the intensities of inflammatory protein staining in Lutein group were significantly higher than Control group.

### Effects of AST and lutein on ICAM-1, MCP-1, and FKN levels in aqueous humors

**Fig 6:**
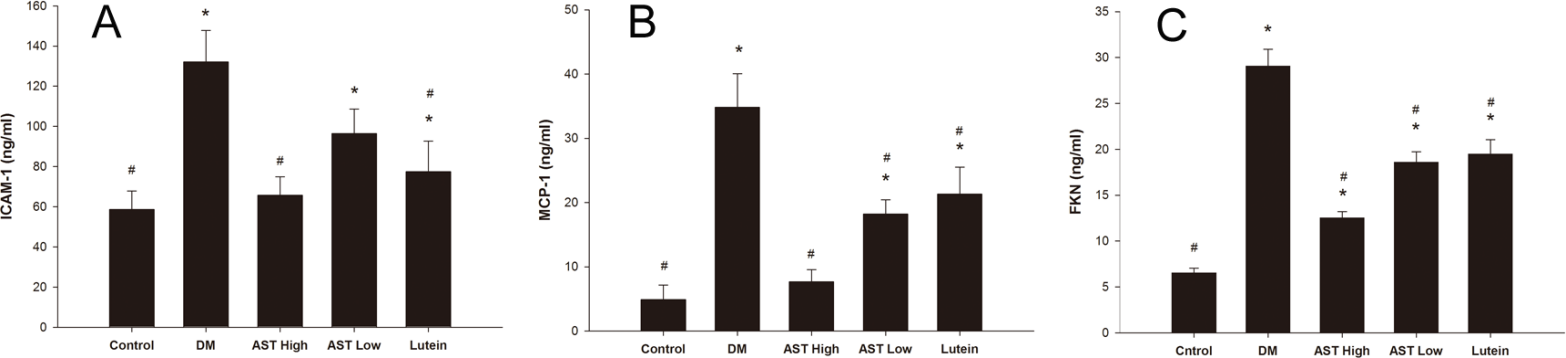
Effect of AST and lutein on ICAM-1, MCP-1, and FKN expression in aqueous humors. Aqueous humor was isolated and pooled from the eyes of rats by each group. ICAM-1, MCP-1, and FKN levels were quantified by 3 repeat ELISA experiments. Data are presented as the mean ± SD. *, *p* < 0.05 versus the Control group; #, *p* < 0.05 versus the Diabetes group.

We measured the concentrations of ICAM-1, MCP-1, and FKN in aqueous via ELISA (Fig 6). Groups treated with high or low dose of AST significantly reduced MCP-1 and FKN in the aqueous humors (*p <* 0.05). In addition, ICAM-1 levels were significantly decreased in the AST High and Lutein groups (*p* < 0.05).

### Expression of antioxidant defense enzyme mRNA levels in retinas

**Fig 7:**
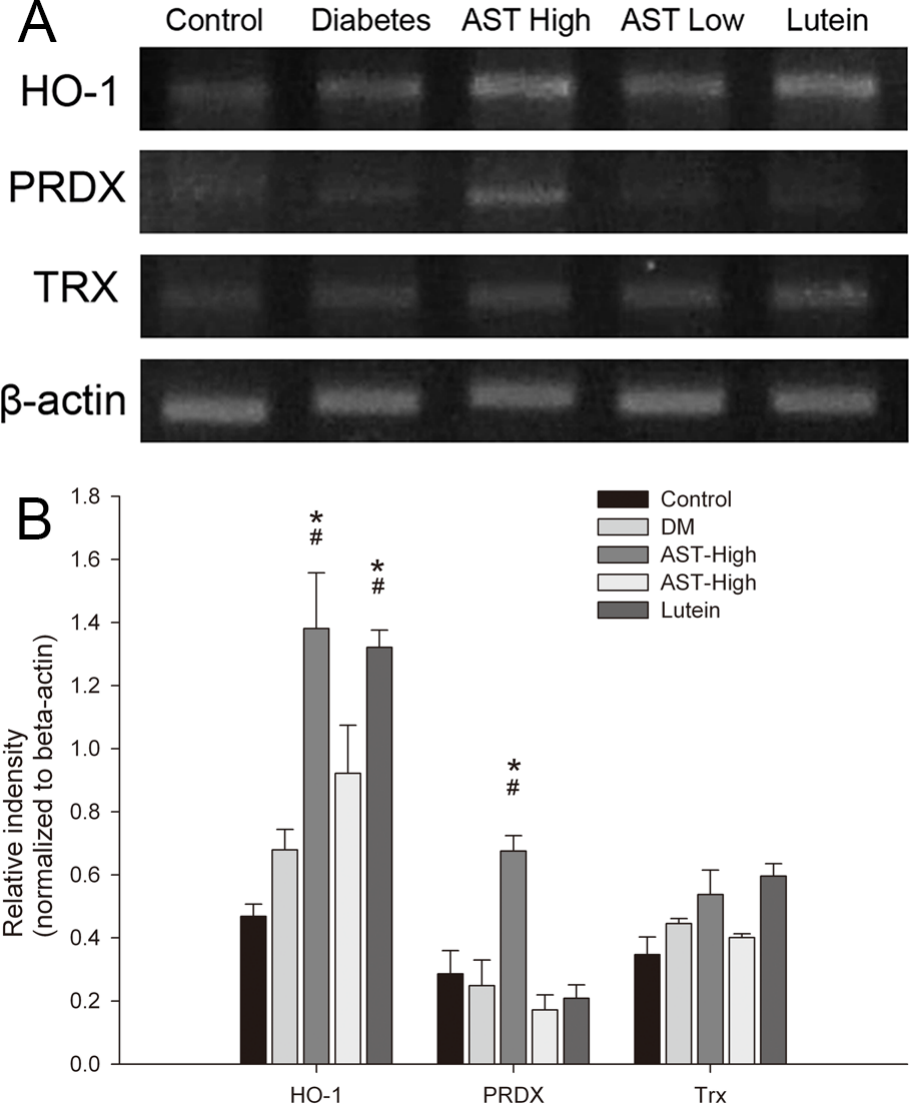
mRNA levels of antioxidant defense enzymes in retinas. RNA was isolated from the retinas of rats by each group. (A) RNA was subjected to RT-PCR using gene-specific primers for HO-1, PRDX, Trx, and β-actin. (B) mRNA expression of HO-1, PRDX, and Trx, normalized to the expression of β-actin. Data are presented as the mean ± SD. *, *p* < 0.05 versus the Control group; #, *p* < 0.05 versus the Diabetes group.

We measured mRNA levels of hemeoxygenase-1 (HO-1), peroxiredoxin (PRDX), and thioredoxin (Trx) by semi-quantified RT-PCR (Fig 7A). The retinas of the Diabetes group contained slightly higher levels of HO-1 and Trx mRNA than the Control group, but they did not reach the significant level. However, the retinas of AST high group had significantly increased levels of HO-1 and PRDX mRNA than Diabetic and Control groups (*p <* 0.05). Lutein also significantly increased HO-1 mRNA than Diabetic and Control groups (*p <* 0.05), but neither AST nor lutein treatment groups affected Trx mRNA expression (Fig 7B).

### Effect of AST and lutein on NF-κB activity in retinas

**Fig 8:**
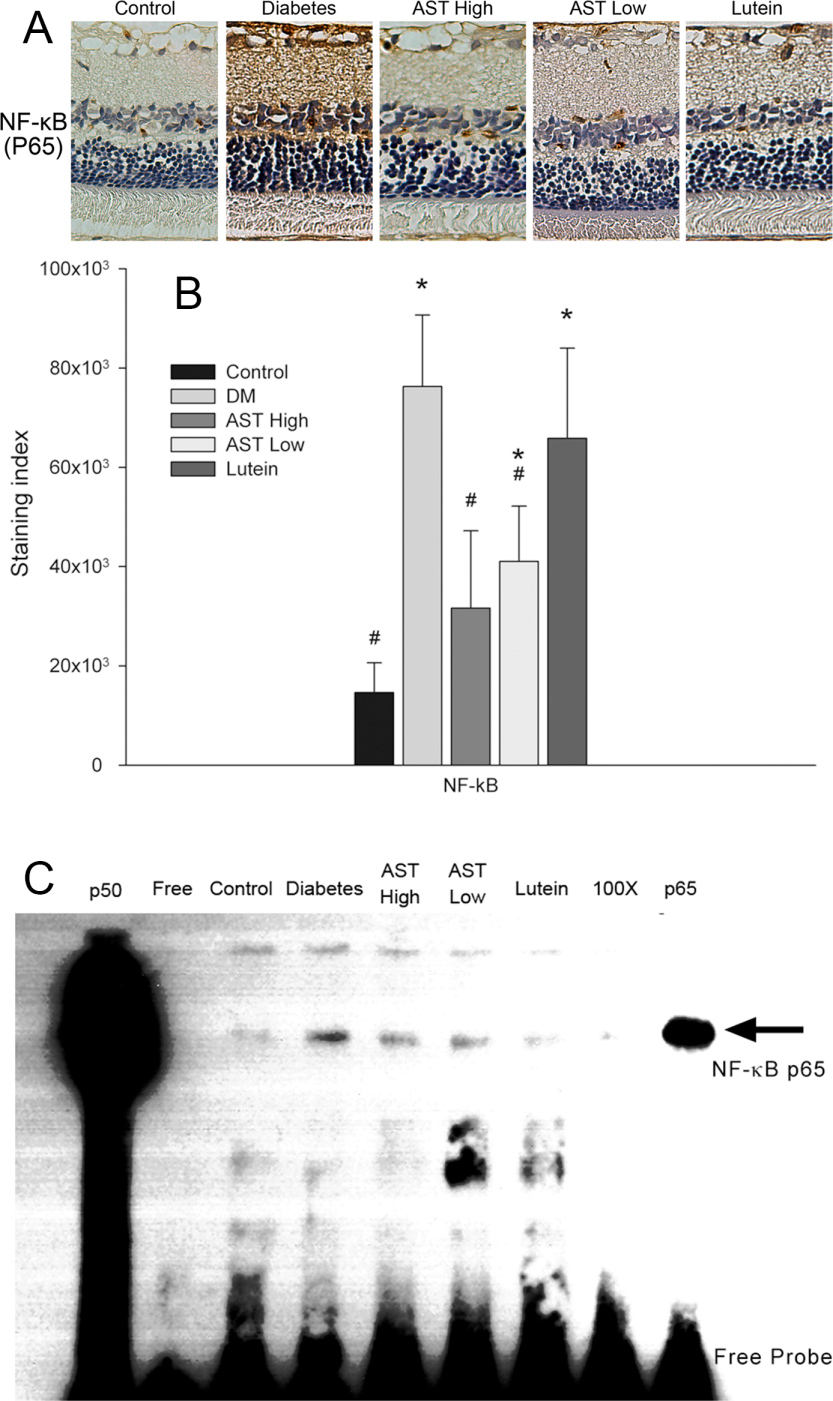
Effect of AST and lutein on NF-κB activity in retinas. (A) Photomicrographs of IHC staining for NF-κB p65 in retinal sections from animals treated as described in Fig 1. (B) The images in (A) were analyzed with Image-Pro software and staining intensity was quantified. Data are presented as the mean ± SD. *, *p* < 0.05 versus the Control group; #, *p* < 0.05 versus the Diabetes group. (C) Nuclear proteins were prepared from the retinas of rats treated as described in Fig 1. EMSA was performed by incubation of extracts with a biotinylated oligonucleotide containing an NF-κB consensus sequence. *Lane 1*: p50 subunit of NF-κB; *lane 2*: free probe (FP); *lane 3*: control rats; *lane 4*: diabetic rats; *lane 5*: diabetic rats treated with high dose astaxanthin (3.0mg/kg/day); *lane 6*: diabetic rats treated with low dose astaxanthin (0.6mg/kg/day); *lane 7*: diabetic rats treated with lutein (0.5mg/kg/day); *lane 8*: 100–fold molar excess of unlabeled NF-κB probe, and *lane 9*: p65, biotinylated probe with anti-p65 antibody.

Retinas from the Control group showed a low level of NF-κB p65 staining in the inner retinas (Fig 8A) and it was markedly increased following diabetes induction. Notably, NF-κB p65 staining was significantly reduced in the retinas of the AST High and AST Low groups (*p <* 0.05; Fig 8B). In Fig 8C, NF-κB activation was assessed by EMSA. Nuclear proteins extracted from the retinas of the Diabetes group showed increased NF-κB–DNA binding activity, and the binding activity was reduced in extracts from rats treated with AST or lutein.

## Discussion

AST is a member of the xanthophyll family of oxygenated carotenoid derivatives. AST is well-known as a powerful free radical scavenger and an excellent anti-inflammatory agent that suppresses proinflammatory cytokine and chemokine expression [21, 29]. The molecule has unique chemical properties derived from its distinctive molecular structure, which includes two hydroxyl groups, two carbonyl groups, and 11 conjugated ethylenic double bonds. The polyene system endows AST with unique chemical properties and light-absorption characteristics [38]. The hydroxyl and keto moieties present on each ionone ring explain the ability of AST to be esterified, as well as the more polar nature and higher antioxidant activity of AST compared with other carotenoids [39]. Nakajuma and colleagues reported that AST was neuroprotective and prevented damage to retinal ganglion cells [29]. Izumi-Nagai et al. have indicated that the anti-inflammatory and antioxidant effect of AST in an animal model of laser-induced choroidal neovascularization is mediated by inhibition of NF-κB pathway activation [40]. AST has been used in the treatment of cardiovascular disease [41], ischemic brain damage [42], cataracts [43], diabetes [44], and diabetic nephropathy [45, 46]. However, the mechanism by which AST reduces STZ-induced diabetic retinopathy in rats has remained unclear. In a previous study, Speranza et al. demonstrated AST reduced oxidative induced proinflammatory cytokines by inhibiting NF-κB expression in U937 cells [47]. Izumi-Nagai et al. showed AST treatment could inhibit NF-κB activation, subsequent downregulation of inflammatory molecules and decreasing macrophage infiltration and then AST further suppressed choroid neovascularization development in mice [40]. Suzuki et al. also proved that AST could oppose rat endoxin-induced uveitis by inhibiting the NF-κB signaling pathway [48]. In our present study, we demonstrated that AST reduced oxidative stress, reduced expression of inflammatory mediators, increased antioxidant enzymes (HO-1 and PRDX) in the retina of diabetic rats and AST significantly preserved the retinal architecture and function. These protective effects might be as well as related to its inhibitory effect on the NF-κB activation.

A strong correlation between oxidative stress and the development of diabetic retinopathy has been confirmed [3–5]. Diabetes increases oxidative stress and ROS levels in the retina, and the induction of antioxidant enzymes is insufficient to prevent retinal damage. Yeh and colleagues reported that ROS levels in vitreous fluid may correlate positively with the severity of diabetic retinopathy [49]. It is likely that increased ROS and oxidative stress causes tissue injury through peroxidation of DNA, lipids, proteins, and carbohydrates [50], with the concomitant production of oxidative biomarkers such as 8-OHdG, nitrotyrosine, carbonylated proteins [51], acrolein, and lipid peroxides [17]. In our study, diabetes significantly increased retinal expression of the oxidation products, 8-OHdG, nitrotyrosine, and acrolein. These results confirmed that diabetes increases oxidative stress in the retina and results in the accumulation of peroxidation products.

An imbalance between oxidants and antioxidants plays a critical role in the development and progression of diabetic retinopathy. Several antioxidant systems have been suggested to be involved. HO-1, a heat shock protein, is extremely sensitive to both oxidative and cellular stress and is acutely upregulated in diabetes [52, 53]. HO-1 catalyses the conversion of heme to biliverdin with the release of carbon monoxide and free iron. Many in vitro, in vivo, and epidemiological studies have shown that HO-1 has protective properties against cardiovascular disease [54]. PRDXs, a family of stress-response antioxidants, can detoxify ROS and thus provide cytoprotection from internal and external oxidative stress [55]. Trx also decreases ROS levels [56]. Trx participates in a variety of redox reactions, such as reversible oxidation of proteins and regulation of transcription factors and apoptosis [57, 58]. Our present study demonstrated antioxidants enzyme levels were not significantly elevated in the diabetic animals. (Fig 7) The maintenance of redox homeostasis in diabetes requires that antioxidant enzyme levels must be elevated to achieve the oxidant–antioxidant balance. AST and lutein may significant promote HO-1 mRNA expression, and high dose AST would elevate PRDX mRNA level. But neither AST nor lutein could increase Trx production.

ROS contribute to inflammatory responses by stimulating the activity of transcription factors NF-κB [45]. In our study, NF-κB p65 expression and activity was shown by IHC and EMSA that it was significantly increased in diabetic retina. The results were consistent with our hypothesis that oxidative stress associated with diabetes would activate NF-κB in the retina. In addition, NF-κB is known to bind to the promoters and increase the transcription of the adhesion molecule ICAM-1 and the chemokines MCP-1 and FKN, all of which are associated with inflammation in diabetic retinopathy [59–62]. These inflammatory molecules could induce vascular endothelial cell damage, increase vascular permeability, and cause cytokine release, which lead to angiogenesis in diabetic retinopathy.

Our results indicate that AST and lutein reduce the generation of oxidation products, including 8-OHdG, nitrotyrosine, and acrolein, in the retinas, suggesting that they inhibit the development of diabetic retinopathy by reducing oxidative damage to DNA, proteins, and lipids. We showed animals fed AST had significantly increased retinal levels of HO-1, and PRDX mRNA, consistent with an advancement of the antioxidant enzyme system capabilities. In addition, our study showed AST and lutein could prevent further retinal damage from oxidative stress and preserve retinal functions. Our results are similar to those obtained by Kowluru et al. in the rat model of diabetes. They demonstrated that micronutrients that were shown to reduce retinal abnormalities in the major Age-Related Eye Disease Study (AREDS) (50 mg/kg of ascorbic acid; 0.5 g/kg of vitamin E; 1.5 mg/kg of beta carotene; 8 mg/kg of zinc oxide; and 0.2 mg/kg of copper oxide) could prevent capillary degeneration in the retinal vasculature of diabetes rats. Notably, the AREDS-based micronutrients increased antioxidant enzyme expression and decreased nitric oxide synthase expression [63]. However, the effects of AREDS-based micronutrients in preventing the deterioration of diabetic retinopathy will require additional investigation to clarify the mechanisms and pathways by which these antioxidants act in the retina of diabetics.

In our study, histological examination of retina showed significant morphological damage and retinal ganglion cell loss in diabetic rats. We displayed not only the preservation of histological and functional outcomes of AST in diabetic rats but the effects of AST on oxidative and inflammatory pathways associated with diabetic retinopathy. Our results suggest that AST has the potential to inhibit the development of diabetic retinopathy by reducing oxidative stress, increasing antioxidant enzymes, inhibiting NF-κB activity, and reducing the expression of downstream inflammatory mediators. AST may thus be a beneficial nutritional supplement to halt the progression of diabetic retinopathy and prevent vision loss in patients with diabetes.
